# NADPH Oxidase Limits Innate Immune Responses in the Lungs in Mice

**DOI:** 10.1371/journal.pone.0009631

**Published:** 2010-03-16

**Authors:** Brahm H. Segal, Wei Han, Jennifer J. Bushey, Myungsoo Joo, Zahida Bhatti, Joy Feminella, Carly G. Dennis, R. Robert Vethanayagam, Fiona E. Yull, Maegan Capitano, Paul K. Wallace, Hans Minderman, John W. Christman, Michael B. Sporn, Jefferson Chan, Donald C. Vinh, Steven M. Holland, Luigina R. Romani, Sarah L. Gaffen, Michael L. Freeman, Timothy S. Blackwell

**Affiliations:** 1 Department of Medicine, Roswell Park Cancer Institute, Buffalo, New York, United States of America; 2 Department of Immunology, Roswell Park Cancer Institute, Buffalo, New York, United States of America; 3 School of Medicine and Biomedical Sciences, University of Buffalo, Buffalo, New York, United States of America; 4 Department of Medicine, Vanderbilt University School of Medicine, Nashville, Tennessee, United States of America; 5 Division of Applied Medicine, School of Oriental Medicine, Pusan National University, Yangsan, Korea; 6 Department of Cancer Biology, Vanderbilt University School of Medicine, Nashville, Tennessee, United States of America; 7 Department of Pathology, Roswell Park Cancer Institute, Buffalo, New York, United States of America; 8 Section of Pulmonary, Critical Care, and Sleep Medicine, University of Illinois at Chicago, Chicago, Illinois, United States of America; 9 Department of Pharmacology and Toxicology, Dartmouth Medical School, Hanover, New Hampshire, United States of America; 10 Department of Pathology, University of Southern California School of Medicine, Irvine, California, United States of America; 11 Laboratory of Clinical Infectious Diseases, National Institute of Allergy and Infectious Disease, National Institutes of Health, Bethesda, Maryland, United States of America; 12 Department of Experimental Medicine, University of Perugia, Perugia, Italy; 13 Division of Rheumatology and Clinical Immunology, University of Pittsburgh, Pittsburgh, Pennsylvania, United States of America; 14 Department of Radiation Oncology, Vanderbilt University School of Medicine, Nashville, Tennessee, United States of America; 15 Department of Cell and Developmental Biology, Vanderbilt University School of Medicine, Nashville, Tennessee, United States of America; 16 Department of Veterans Affairs, Nashville, Tennessee, United States of America; University of Alabama-Birmingham, United States of America

## Abstract

**Background:**

Chronic granulomatous disease (CGD), an inherited disorder of the NADPH oxidase in which phagocytes are defective in generating superoxide anion and downstream reactive oxidant intermediates (ROIs), is characterized by recurrent bacterial and fungal infections and by excessive inflammation (e.g., inflammatory bowel disease). The mechanisms by which NADPH oxidase regulates inflammation are not well understood.

**Methodology/Principal Findings:**

We found that NADPH oxidase restrains inflammation by modulating redox-sensitive innate immune pathways. When challenged with either intratracheal zymosan or LPS, NADPH oxidase-deficient p47*^phox−/−^* mice and gp91*^phox^*-deficient mice developed exaggerated and progressive lung inflammation, augmented NF-κB activation, and elevated downstream pro-inflammatory cytokines (TNF-α, IL-17, and G-CSF) compared to wildtype mice. Replacement of functional NADPH oxidase in bone marrow-derived cells restored the normal lung inflammatory response. Studies *in vivo* and in isolated macrophages demonstrated that in the absence of functional NADPH oxidase, zymosan failed to activate Nrf2, a key redox-sensitive anti-inflammatory regulator. The triterpenoid, CDDO-Im, activated Nrf2 independently of NADPH oxidase and reduced zymosan-induced lung inflammation in CGD mice. Consistent with these findings, zymosan-treated peripheral blood mononuclear cells from X-linked CGD patients showed impaired Nrf2 activity and increased NF-κB activation.

**Conclusions/Significance:**

These studies support a model in which NADPH oxidase-dependent, redox-mediated signaling is critical for termination of lung inflammation and suggest new potential therapeutic targets for CGD.

## Introduction

The lung is an interface where inhaled microbes and antigens interact with host defense cells. Pathogen recognition receptors (PRRs) such as toll-like receptors (TLRs) sample microbial motifs and initiate signaling that may result in NADPH oxidase activation. NADPH oxidase activation requires translocation of the cytoplasmic subunits p47*^phox^*, p67*^phox^*, and p40*^phox^* and rac to the membrane-bound flavocytochrome consisting of gp91*^phox^* and p22*^phox^* (phox, phagocyte oxidase). NADPH oxidase activation leads to generation of superoxide anion and downstream reactive oxidant intermediates (ROIs) and activation of neutrophil antimicrobial proteases [1], [2], [3]. ROIs have been implicated in the pathogenesis of lung diseases through several mechanisms, including cellular injury and NF-κB activation [4], [5].

Chronic granulomatous disease (CGD) is an inherited disorder of NADPH oxidase characterized by life-threatening bacterial and fungal infections and by abnormally exuberant inflammatory responses (e.g., inflammatory bowel disease) [6]. “Mulch pneumonitis” is a hyper-inflammatory response in CGD patients to fungal pneumonia[7]. Studies in CGD patients [8], [9], [10], [11] and mouse models [12], [13], [14], [15], [16], [17], [18], [19] point to excessive inflammation in CGD resulting from intrinsic defect(s) in immune regulation.

We evaluated whether NADPH oxidase activity would counterbalance the immediate pro-inflammatory events that follow PRR signaling by interacting with redox-sensitive pathways to dampen inflammation. Using p47*^phox−/−^* [20] and gp91*^phox^-*deficient [21] mice that lack NADPH oxidase function, we show that NADPH oxidase limits inflammation by attenuating the pro-inflammatory transcription factor NF-κB and by activating Nrf2, an anti-inflammatory transcription factor. Our studies demonstrate pharmacological activation of Nrf2 as a potential therapeutic strategy in CGD. This work identifies NADPH oxidase as a critical regulator of innate immunity and provides novel understanding of mechanisms that regulate lung inflammation.

## Results and Discussion

### NADPH Oxidase Down-Regulates Zymosan-Induced Lung Inflammation

We asked whether NADPH oxidase, which is activated by bacterial and fungal pathogens, would have a role in restraining lung inflammation induced by microbial motifs. We selected microbial products rather than live bacterial or fungal pathogens to specifically evaluate NADPH oxidase as a regulator of inflammation independently of its antimicrobial function; thus a limitation of these studies is that they intentionally do not encapsulate the complexity of *in vivo* infection models. Since zymosan is a pro-inflammatory yeast cell wall product comprised predominantly of particulate β-glucan that ligates TLR2 and is a potent activator of NADPH oxidase via dectin-1 signaling [22], we used intratracheal zymosan to induce sterile lung inflammation. Lungs were harvested between 6 hours to 25 days after a single administration of zymosan.

**Figure 1:**
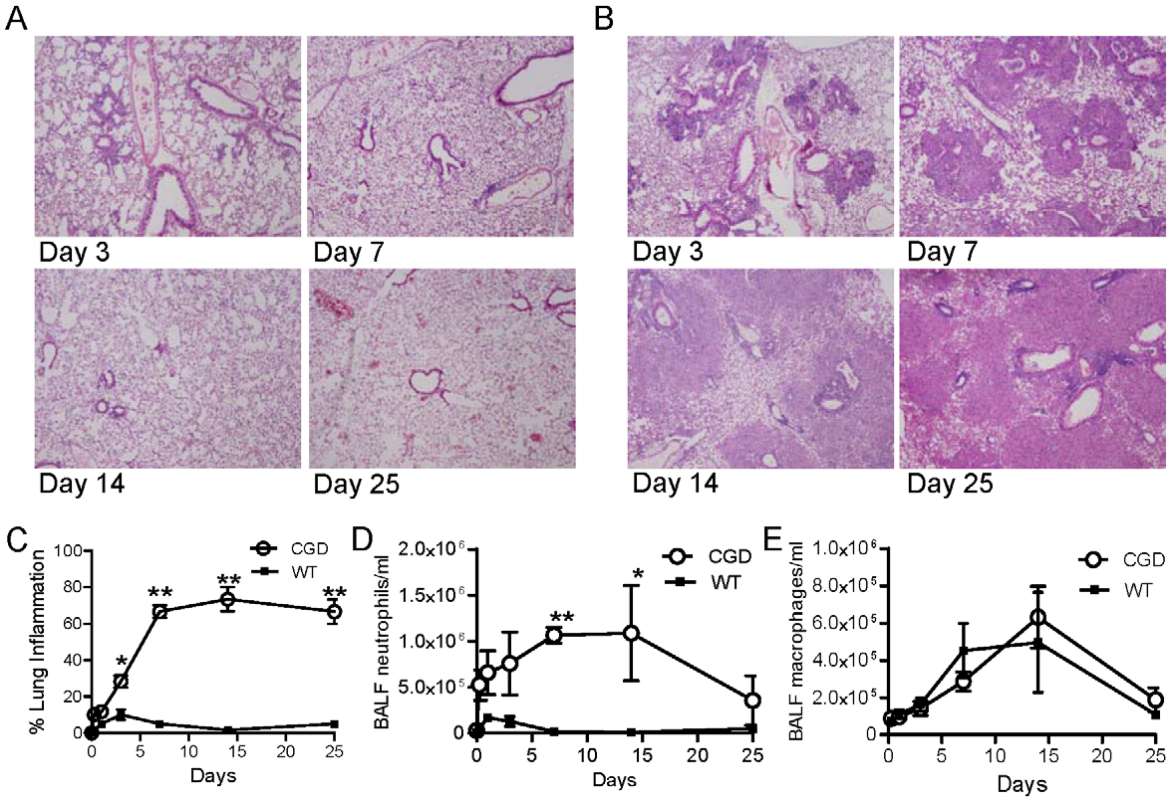
p47*^phox−/−^* mice develop increased zymosan-induced lung inflammation compared to wildtype (WT) mice. Representative lung histology of WT (A) and p47*^phox−/−^* (CGD) mice (B) at days 3, 7, 14, 25 after i.t. zymosan. All slides are H&E stained, 20x, and are representative of at least 3 mice per genotype per time point. C) Percent of lung parenchyma with consolidation or granulomatous inflammation. Bronchoalveolar lavage fluid (BALF) neutrophil (D) and macrophage (E) concentrations. 2-way ANOVA showed a significant difference between genotypes in % lung inflammation (p<0.0001) and BALF neutrophil concentration (p<0.0001), with significant differences at the indicated time points by Boneferroni post-test. *, p<0.01; **, p<0.001).

Wildtype (WT) mice developed mild peribronchial inflammation (Figure 1A). In contrast, p47*^phox−/−^* (CGD) mice developed a robust and persistent inflammatory response following zymosan administration (Figure 1B). The kinetics of zymosan-induced histological lung inflammation in WT and CGD mice demonstrate the dramatic difference between genotypes (Figure 1C). The early inflammatory response in p47*^phox−/−^* mice was principally neutrophilic (days 1 to 3). Well-defined pyogranulomatous lesions consisting of foci of neutrophils surrounded by lymphohistiocytic infiltrates were present on day 7 after zymosan administration. On days 14 and 25, the neutrophilic component of the infiltrates was reduced compared to earlier time points and the inflammation was predominantly lymphohistiocytic. Bronchoalveolar lavage fluid (BALF) cytology showed persistence of predominantly neutrophilic inflammation through day 14 after zymosan administration in p47*^phox−/−^* mice, whereas BALF neutrophilic leukocytosis in WT mice normalized by day 7 (>90% macrophages; Figure 1D and E). These results demonstrate that resolution of lung inflammation is impaired by loss of NADPH oxidase activity.

### NADPH Oxidase Regulates Pro-Inflammatory Cytokine Production and NF-κB Activation

**Figure 2:**
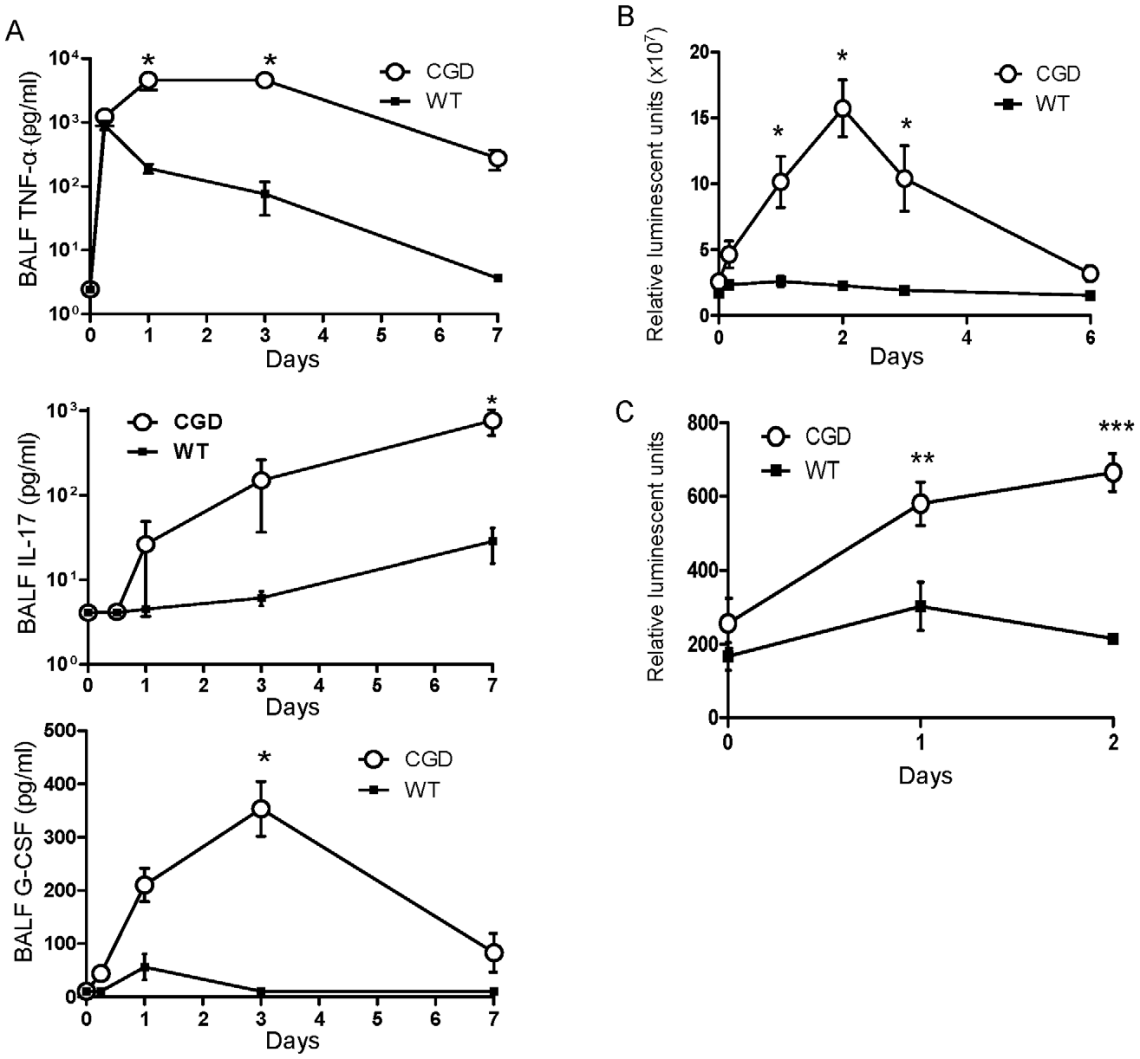
Intratracheal zymosan treatment results in higher levels of pro-inflammatory cytokines and NF-κB activation in lungs of p47*^phox−/−^* mice. A) BALF levels of TNF-α, IL-17, and G-CSF in wild type (WT) and p47*^phox−/−^* (CGD) mice administered i.t. zymosan. Note that the Y-axes in the TNF-α and IL-17 graphs are in log-scale. The interaction of genotype (p47*^phox−/−^* vs. WT) and time was assessed by 2-way ANOVA and was significant for each of the 3 cytokines (p<0.001). Bonferroni post-test was used to test for significance at each time point (*, p<0.05). B) Whole lung NF-κB activation measured by bioluminescence imaging over the chest after i.v. luciferin in NF-κB reporter mice (p47*^phox−/−^*/HLL and WT/HLL). C) NF-κB dependent luciferase activity in bone marrow-derived macrophages (BMDMs) from p47*^phox−/−^*/HLL and WT/HLL mice after *in vitro* stimulation with zymosan (20 µg/ml). For (B) and (C), 2-way ANOVA indicated p<0.0001 between genotypes with significant differences at the indicated time points by Bonferroni post-test. *, p<0.05; **, p<0.01; ***, p<0.001).

We measured a number of pro-and anti-inflammatory cytokines present in BALF at days 1, 3 and 7 after i.t. zymosan treatment. TNF-α, IL-17, and G-CSF levels in BALF were increased in p47*^phox−/−^* mice compared to WT mice (Figure 2A). IL-1β concentration was increased in p47*^phox−/−^* mice on day 3, but not at other time points, whereas BALF levels of interferon-γ, IL-2, IL-4, IL-6, IL-10, IL-12, KC, MCP-1, MIP-2, and TGF-β were similar between WT and p47*^phox−/−^* mice (data not shown).

Since NF-κB induces the expression of several pro-inflammatory genes and is influenced by redox status, we evaluated whether NF-κB activation was differentially regulated in p47*^phox−/−^* and WT mice. Using p47*^phox−/−^*/HLL and WT/HLL mice that express luciferase under the control of an NF-κB dependent promoter [23], we measured *in vivo* luciferase expression using bioluminescence imaging. Zymosan-induced whole lung NF−κB activation was significantly augmented in p47*^phox−/−^*/HLL compared to WT/HLL mice. NF-κB-dependent luciferase expression peaked at 2 days post-zymosan in p47*^phox−/−^*/HLL mice (6.3-fold above baseline), whereas luciferase activity was not increased above baseline in WT/HLL mice at this time point (Figure 2B).

Consistent with these *in vivo* findings, zymosan treatment also resulted in increased NF-κB activation in isolated p47*^phox−/−^* bone marrow-derived macrophages (BMDMs) compared to WT BMDMs (Figure 2C). As expected, WT BMDMs had augmented superoxide production in response to zymosan and PMA as measured by chemiluminescence, whereas CGD macrophages failed to do so (data not shown).

Whereas whole lung NF-κB activation peaked at day 2 and returned to unstimulated levels by day 6 in p47*^phox−/−^*/HLL mice, histological lung inflammation continued to progress (Figure 1), arguing that NF-κB activation in whole lungs is not simply a non-specific marker of inflammation. These findings are consistent with NF-κB activation having a role in initiating the early inflammatory cascade while down-stream cytokines and additional pathways play important roles in maintenance of persistent inflammation in p47*^phox−/−^* mice. *In vitro* data using isolated macrophages further support the role of NADPH oxidase as a negative regulator of zymosan-induced NF-κB activation.

We examined whether the inflammatory dysregulation in p47*^phox−/−^* mice after zymosan could be explained by differences in surface expression of PRRs, binding of zymosan, or impaired clearance of β-glucan. Using fluorescently tagged zymosan (Alexa Fluor 488), no difference in zymosan binding or phagosomal uptake in p47*^phox−/−^* and WT macrophages occurred. Surface expression of TLR2 (anti-TLR2; eBioscience, San Diego, CA) and dectin-1 (anti-Dectin-1 mAb, 2A11 was a gift from Gordon Brown, PhD, University of Aberdeen, UK) in unstimulated macrophages and at 15 and 60 minutes after zymosan stimulation was similar between WT and p47*^phox−/−^* cells (data not shown). We also tested whether clearance of β-glucan, the principal component of zymosan, was defective in p47*^phox−/−^* mice. In fact, levels of β-glucan in BALF (measured by Fungitell assay; Associates of Cape Cod, East Falmouth, MA) were similar or greater in WT mice, a finding that may reflect increased clearance as a result of enhanced lung inflammation in p47*^phox−/−^* mice (data not shown). These results point to NADPH oxidase-regulated signaling events accounting for the differences in the inflammatory phenotypes between WT and p47*^phox−/−^* mice, rather than differences at the level of binding to surface receptors.

**Figure 3:**
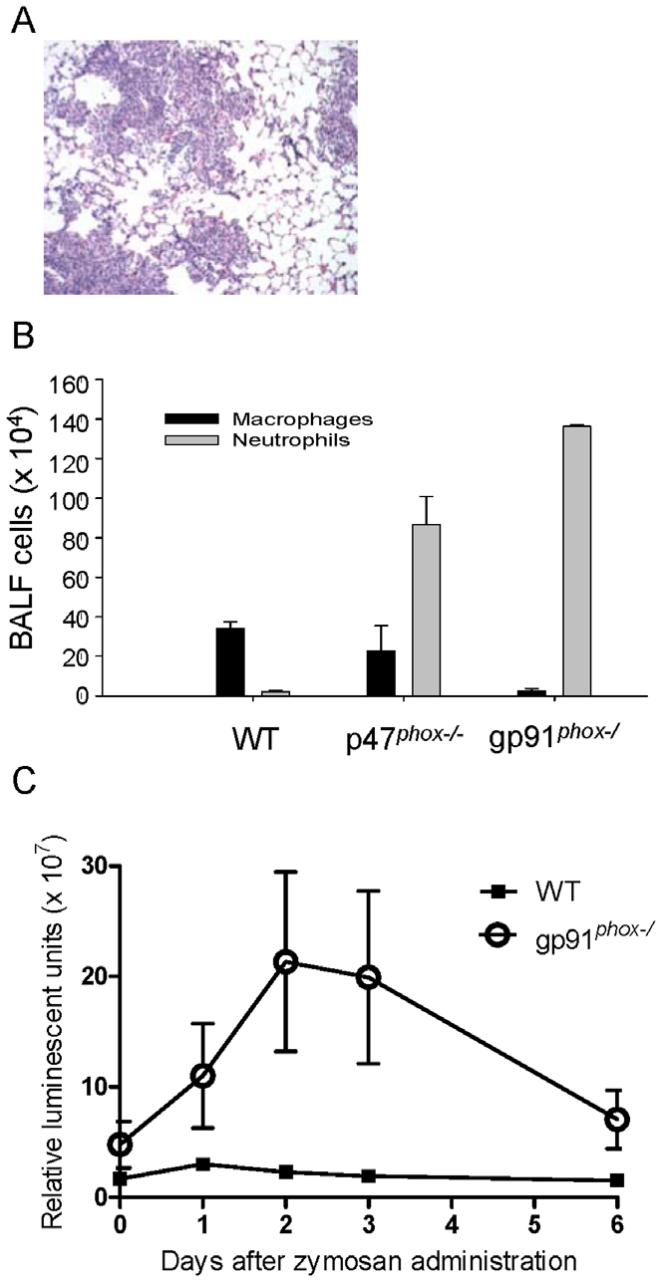
Intratracheal zymosan caused increased lung inflammation and NF-κB activation, in gp91*^phox^-*deficient (X-linked CGD) versus wildtype mice. A) Representative lung section of gp91*^phox^-*deficient mouse 6 days after i.t. zymosan showing extensive inflammation (H&E, 100x). Similarly treated wildtype (WT) mice had no lung inflammation (not shown). B) On day 6 after i.t. zymosan, both NADPH oxidase deficient genotypes (p47*^phox−/−^* and gp91*^phox−/^*) had similar BALF neutrophilic leukocytosis, whereas monocytes predominated in WT BALF. C) gp91*^phox^-*deficient/HLL mice had increased whole lung NF-κB activation compared to WT/HLL mice.

Since it is possible that p47*^phox^* can modulate inflammation independently of NADPH oxidase, we evaluated zymosan-induced lung inflammation in X-linked gp91*^phox−/^* CGD mice [21]. We selected a single time point (6 days). The histological lung inflammatory response and inflammatory cell influx in BALF in gp91*^phox−/^* mice were similar to p47*^phox−/−^* mice, except for a trend towards fewer macrophages in BALF from gp91*^phox−/^* mice at this time point (Figure 3A and B). Similar to p47*^phox−/−^* mice, i.t. zymosan-induced lung NF-κB activation was significantly increased in gp91*^phox−/^* mice compared to similarly treated WT mice (Figure 3C). These consistent findings in two different mouse models support the critical role of NADPH oxidase in down-regulating inflammation as opposed to an individual phox protein functioning independently of NADPH oxidase or an artifact introduced during generation of one of the knockout colonies.

**Figure 4:**
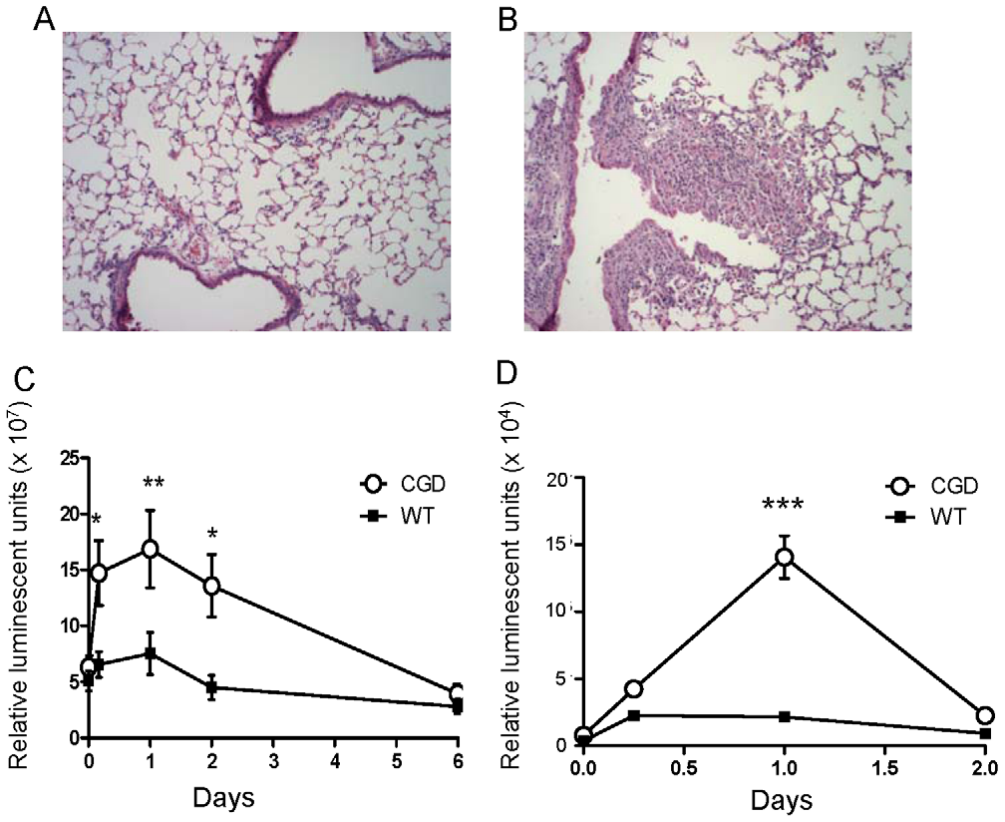
CGD mice develop increased lipopolysaccharide-induced lung inflammation and NF-κB activation compared to wildtype (WT) mice. Wildtype (WT)/HLL and p47*^phox−/−^* /HLL (CGD) mice were administered intratracheal (i.t.) LPS (3 µg/g per mouse). Representative lung histology of a WT/HLL (A) and p47*^phox−/−^*/HLL (B) mouse 6 days after i.t. LPS (H&E, 100x). Minimal to no lung inflammation was present in WT/HLL mice, whereas mixed neutrophilic and lymphohistiocytic infiltrates involving ∼10% of the lung and interstitial edema was present in p47*^phox−/−^*/HLL mouse lungs. Whole lung NF-κB activation (C) was augmented in p47*^phox−/−^*/HLL mice administered i.t. LPS compared to similarly treated WT/HLL mice (2-way ANOVA, p<0.0001, with Bonferroni post-test showing significant differences between genotypes at the indicated time points). D) Isolated p47*^phox−/−^*/HLL bone marrow-derived macrophages had augmented NF-κB activation in response to LPS compared to similarly treated WT/HLL macrophages (2-way ANOVA, p<0.0001). *, p<0.05; **, p<0.01; ***, p<0.001.

We next asked whether ligation of other PRRs would lead to augmented lung inflammation and NF-κB activation in CGD compared to WT mice as did zymosan. Lipopolysachharide (LPS), a bacterial cell wall constituent, ligates CD14/TLR4 and causes a redistribution of NADPH oxidase components in neutrophils that primes the respiratory burst in response to other agents [22], [24]. Similar to zymosan, i.t. treatment with LPS induced greater lung inflammation (Figure 4A and B) and NF-κB activation (Figure 4C) in p47*^phox−/−^* compared to WT mice. In addition, *in vitro* LPS stimulation induced greater NF-κB activation in isolated p47*^phox−/−^* macrophages compared to WT macrophages (Figure 4D). These consistent results using microbial motifs that ligate distinct PRRs underscore a broad role of NADPH oxidase in down-regulating inflammation.

### NADPH Oxidase in Hematopoietic Cells, but Not Lung Stromal Cells, Is Essential to Restrain Zymosan-Induced Lung Inflammation

**Figure 5:**
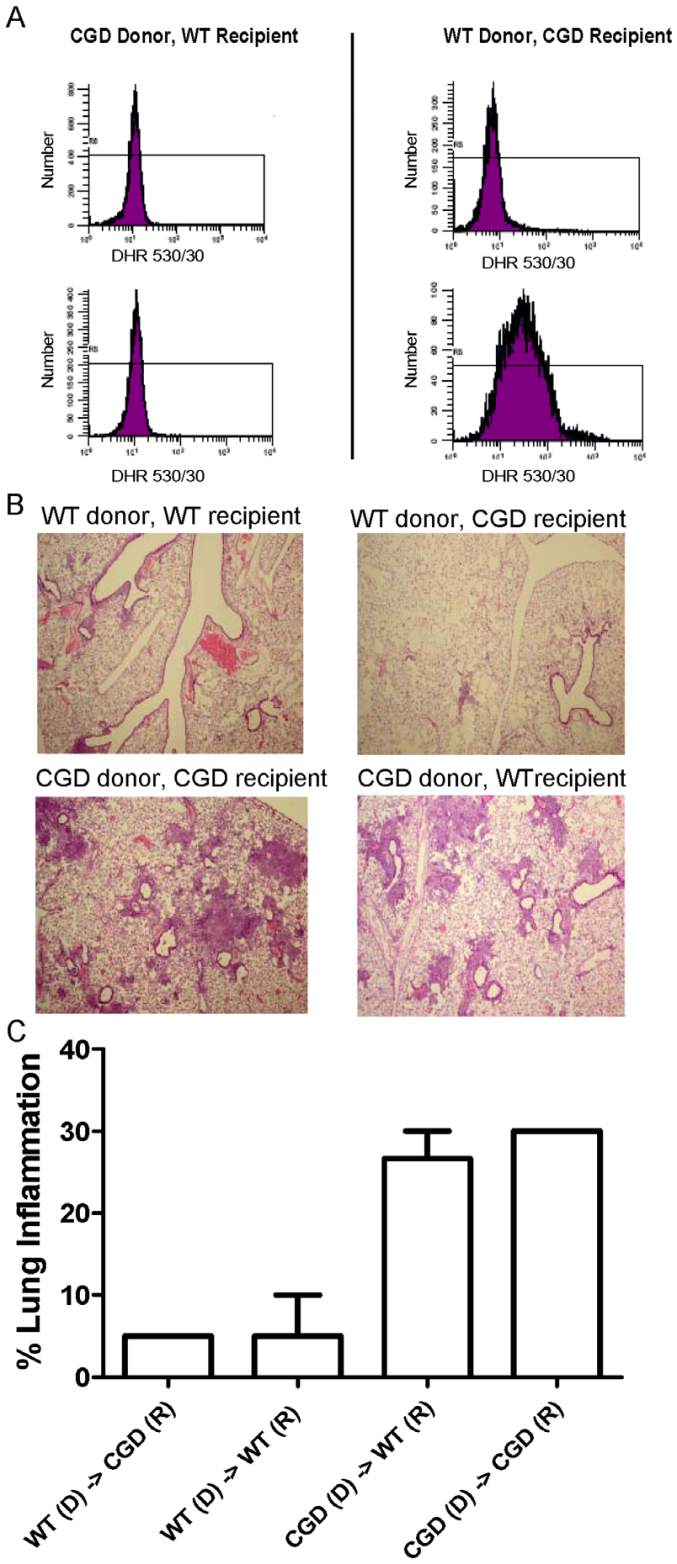
Bone marrow chimera experiments demonstrate that NADPH oxidase in hematopoietic cells, but not lung stromal cells, is required to restrain zymosan-induced lung inflammation. Wild type (WT) and p47*^phox−/−^* (CGD) mice were administered myeloablative total body irradiation, and rescued with marrow-derived WT or p47*^phox−/−^* donor cells. The transplants were as follows: WT donor/CGD recipient (n = 3); CGD donor/WT recipient (n = 3); WT donor/WT recipient (n = 2); CGD donor/CGD recipient (n = 2); the latter two transplants were performed as specificity controls for artifacts introduced by transplantation. Recipient mice were administered i.t. zymosan at 31 days after transplant. Seven days later, peripheral blood and lungs were harvested. A) NADPH oxidase activity in peripheral neutrophils of transplanted mice was evaluated using the fluorescent probe, dihydrorhodamine 123 (DHR). In a representative experiment, neutrophils were unstimulated (top row) or stimulated with PMA (100 ng/ml) (bottom row) to activate NADPH oxidase. Hydrogen peroxide, a metabolite of NADPH oxidase activation, activates DHR fluorescence. In all transplants, the donor genotype determined NADPH oxidase competence. B) Representative lung histology (H&E, 40x) and C) percent of lung parenchyma with consolidation or granulomatous inflammation in transplanted mice at day 7 after i.t. zymosan administration. Percent lung inflammation was significantly greater in transplanted mice with CGD compared to wildtype donors (abbreviated, “D” in the figure) (unpaired t-test, p<0.0001), whereas recipient (abbreviated, “R” in the figure) genotype had no effect on the inflammatory response.

Since NADPH oxidase isoforms exist in several non-hematopoietic cells and have diverse physiological functions [25], we evaluated whether NADPH oxidase in hematopoietic cells is required to restrain inflammation by generating mouse chimeras in which either the hematopoietic or lung stromal cell population harbor a functional NADPH oxidase. In all bone marrow chimeras, the donor genotype determined NADPH oxidase competence in circulating neutrophils, confirming that the transplants were successful (Figure 5A). The lung inflammatory response to zymosan was entirely dependent on the donor genotype (Figure 5B and C). These results show that NADPH oxidase in hematopoietic cells is required to restrain excessive zymosan-induced inflammation, whereas NADPH oxidase in non-hematopoietic lung stromal cells appears to be dispensable.

### NADPH Oxidase Is Required for Zymosan-Induced Nrf2 Activation

Previous studies in cultured cells using a flavoenzyme inhibitor have shown that NADPH oxidase can be an upstream regulator of Nrf2 [26], [27], [28], a redox-sensitive transcription factor that is critical for suppression of inflammatory responses [29]. We asked whether zymosan activates Nrf2 and whether Nrf2 activation is NADPH oxidase-dependent. If so, we reasoned that defective Nrf2 activation could contribute to the hyper-inflammatory phenotype in p47*^phox−/−^* mice.

Typically, Cullin 3 (CUL3) directs the ubiquitination and subsequent proteasome-dependent degradation of Nrf2 [30], [31], [32]. Oxidation or adduction of critical cysteine residues on the adapter protein, Keap1, induces a conformational change that inhibits its ability to bind to CUL3, thereby abrogating Nrf2 ubiquitination and allowing accumulation of transcriptionally active Nrf2 in the nucleus [31], [33]. NADPH oxidase-derived ROIs could activate Nrf2 via oxidation of redox-sensitive cysteine residues on Keap1.

In initial studies, we found that zymosan up-regulates nuclear localization of Nrf2 in RAW264.7 cells by performing western blots from nuclear protein extracts obtained 4 hours after zymosan treatment. We then co-transfected epitope-tagged constructs for Keap1 and CUL3 into RAW 264.7 macrophages and stimulated the cells with zymosan. By co-immunoprecipitation, we found reduced association between Keap1 and CUL3 at 4 hours after zymosan, suggesting that zymosan activates Nrf2 by interfering with Keap1/CUL3 interactions (data not shown).

**Figure 6:**
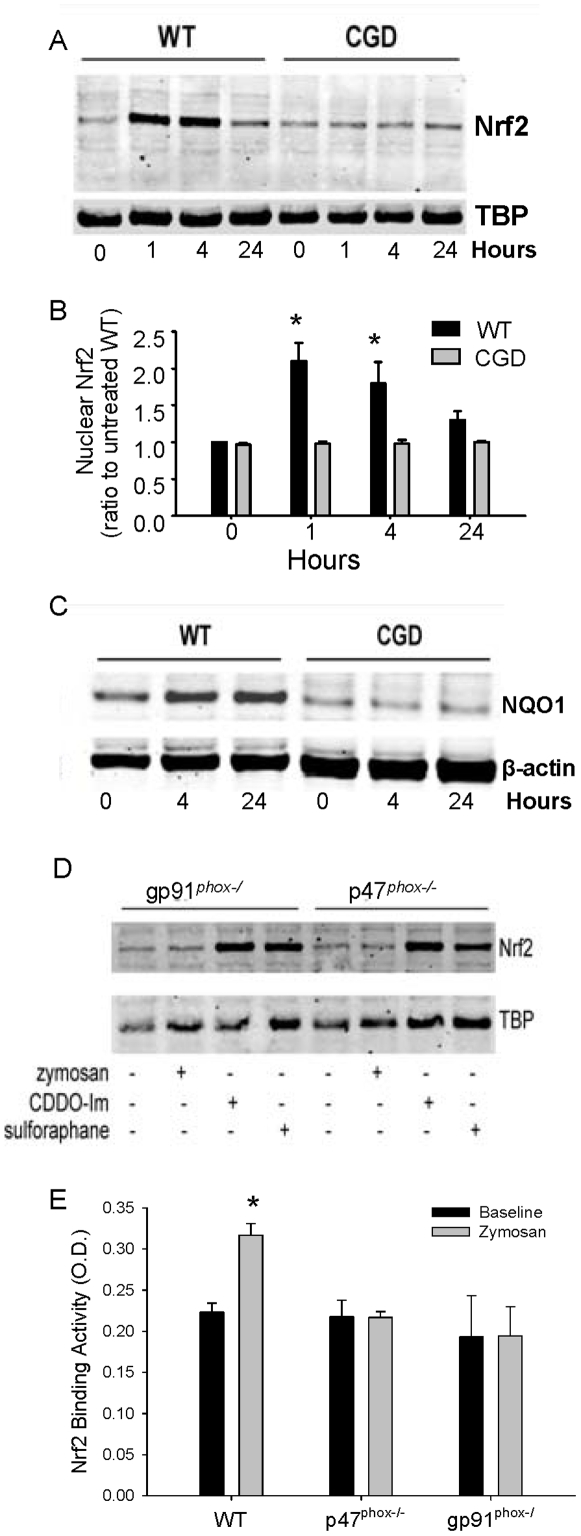
Zymosan-induced Nrf2 nuclear translocation is NADPH oxidase-dependent in isolated macrophages and *in vivo*. Wild type (WT) and p47*^phox−/−^* (CGD) bone marrow-derived macrophages (BMDMs) cultured in DMEM media with 10% serum were stimulated with zymosan (20 µ/ml). A) Western blot for Nrf2 and Tata box binding protein (TBP) in nuclear fractions of BMDMs from p47*^phox−/−^* and WT mice at baseline, 1, 4, and 24 hours after zymosan. B) Densitometry from 3 separate experiments. p<.05 by ANOVA using Tukey post-test. C) Western blot showing expression of NQO1 in cytoplasmic extracts. D) Western blot for nuclear Nrf2 from gp91*^phox−/^* and p47*^phox−/−^* macrophages treated with vehicle, zymosan (20 µg/ml), or the Nrf2 agonist electrophiles, CDDO-Im (1.0 µM) or sulforaphane (50 µM), for 4 hours. E) Nrf2 DNA binding activity of lung nuclear extracts in unstimulated (baseline) mice and 6 days after i.t. zymosan. n = 3−6 mice per group. Lung Nrf2 binding activity in zymosan-stimulated WT mice was significantly greater than that in unstimulated WT mice (student's t-test, p<0.05), whereas zymosan treatment had no effect on lung Nrf2 activation in CGD mice.

To investigate whether Nrf2 activation is impaired in p47*^phox−/−^* mice, we measured Nrf2 nuclear localization in BMDMs stimulated with zymosan. Unstimulated p47*^phox−/−^* and WT macrophages had similar recovery of Nrf2 from nuclear extracts; however, increased nuclear Nrf2 was detected in WT but not in p47*^phox−/−^* macrophages after 1 and 4 hours of zymosan stimulation (Figure 6A and B). The NQO1 promoter contains an antioxidant response element (ARE), the target sequence for Nrf2, and is known to be Nrf2-inducible. We therefore evaluated protein levels of NQO1 in cytoplasmic extracts of p47*^phox−/−^* and WT macrophages following zymosan stimulation as an indirect readout of Nrf2 activation. Consistent with Nrf2 nuclear translocation, NQO1 expression was induced by zymosan in WT, but not in p47*^phox−/−^*, macrophages (Figure 6C).

Since zymosan failed to induce Nrf2 nuclear translocation in p47*^phox−/−^* macrophages, we asked whether well-characterized electrophilic Nrf2 agonists 1-[2-cyano-3-,12-dioxooleana-1,9(11)-dien-28-oyl]imidazole (CDDO-Im) [34] and sulforaphane [35] were capable of activating Nrf2 in p47*^phox−/−^* mouse macrophages. CDDO-Im and sulforaphane were both able to induce Nrf2 nuclear translocation in gp91*^phox−/^* and p47*^phox−/−^* macrophages, whereas zymosan failed to increase Nrf2 translocation above vehicle control (Figure 6D). These *in vitro* studies indicate a stimulus-dependent defect in Nrf2 activation in NADPH oxidase-deficient cells, suggesting that NADPH oxidase-produced ROIs are required for Nrf2 activation following zymosan treatment but are dispensable for electrophile-induced Nrf2 activation.

We then evaluated whether Nrf2 activation was NADPH oxidase-dependent *in vivo*. We measured induction of Nrf2 activity in whole lung nuclear protein extracts from WT, p47*^phox−/−^* and gp91*^phox−/^* mice using an ELISA-based method that measures binding of Nrf2 to its oligonucleotide target. Lung Nrf2 activity was similar at baseline between WT and CGD mice, but was increased after i.t. zymosan only in WT mice (Figure 6E). Taken together, these studies support a role for NADPH oxidase in activating Nrf2 and point to defective Nrf2 activation as a possible mechanism for excessive inflammation in CGD.

### CDDO-Im Attenuates the Zymosan-Stimulated Hyper-Inflammatory Phenotype in p47*^phox−/−^* Mice

**Figure 7:**
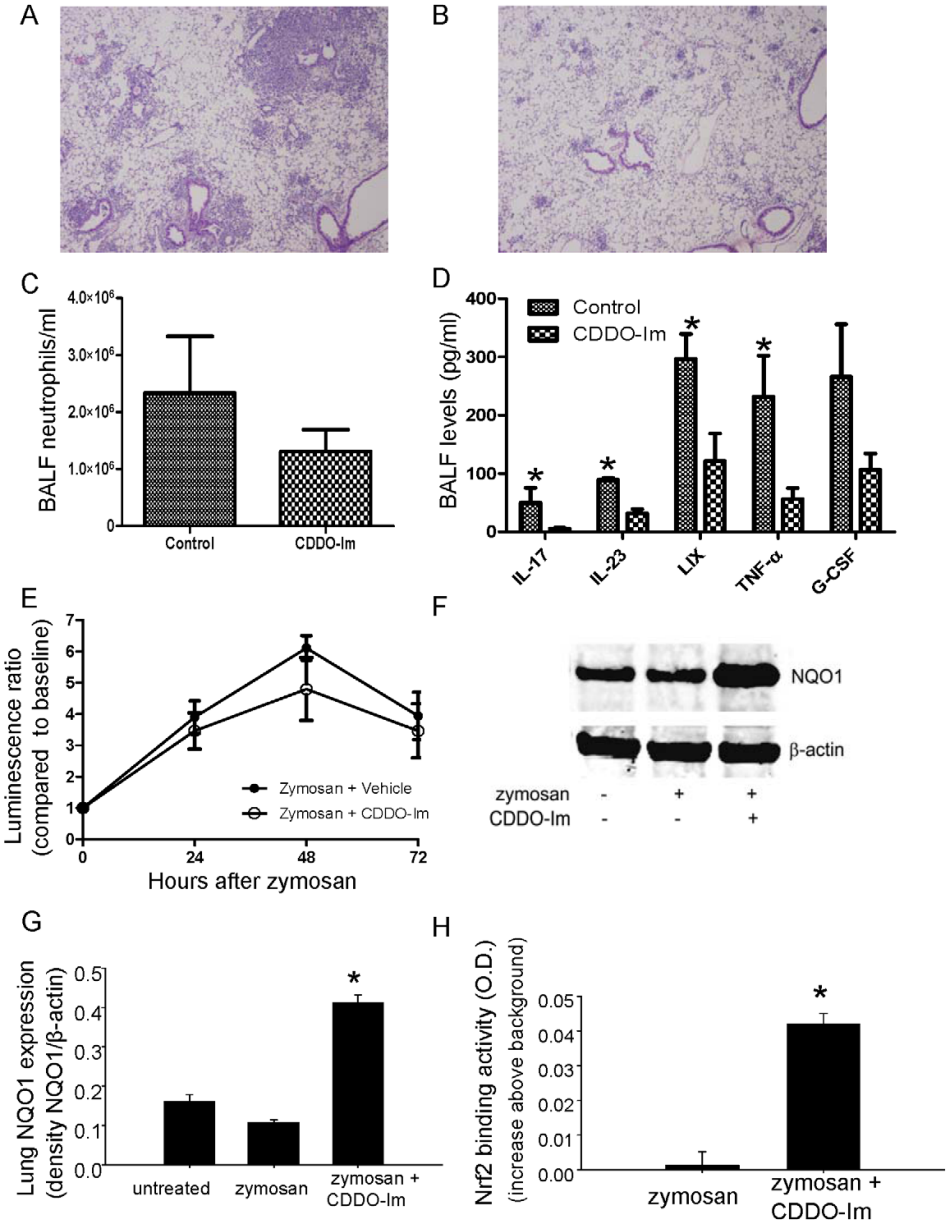
The triterpenoid, CDDO-Im, a Nrf2 inducer, reduces zymosan-induced lung inflammation and pro-inflammatory BALF cytokines in p47*^phox−/−^* mice. CDDO-Im (0.2 mg/mouse by i.p. injection) or vehicle (control) was administered daily to p47*^phox−/−^* mice from day −1 to +2 in relation to i.t. zymosan, and BALF and lungs were harvested on day +3. Representative H&E stained lung sections of p47*^phox−/−^* mice administered zymosan plus vehicle (A) or zymosan plus CDDO-Im (B). Neutrophil (C) and cytokine (D) concentrations were assessed in BALF obtained at day 3 after zymosan treatment. Significant differences were observed for neutrophils (p = 0.03), IL-23 (p = 0.008), IL-17 (p = 0.02), TNF-α (p = 0.02), and LIX (p = 0.03) (Mann-Whitney two-tailed test). E) Lung NF-κB activation, measured by bioluminescence, was similar in p47*^phox−/−^* /HLL mice administered zymosan plus CDDO-Im versus zymosan plus vehicle (Two-way ANOVA, p = NS). F) Representative Western blot of lung homogenates for NQO1 and (G) densitometry (normalized to β-actin) (G) for 3 mice per genotype per treatment (p<.05 by ANOVA using Tukey post-test). Untreated  =  no experimental manipulation; zymosan  =  i.t. zymosan plus i.p. vehicle; zymosan + CDDO-Im  =  i.t. zymosan plus i.p. CDDO-Im. H) Measurement of Nrf2 activity by TransAM™ ELISA from whole lung nuclear protein extracts from p47*^phox−/−^* mice treated with zymosan plus vehicle or zymosan plus CDDO-Im. Results are presented as increase over background O.D. measurement in lung nuclear protein samples from Nrf2^−/−^ mice (p<.05 using unpaired t-test).

To evaluate the role of Nrf2 in the hyper-inflammatory phenotype of p47*^phox−/−^* mice, we tested whether CDDO-Im would attenuate zymosan-induced lung inflammation in these mice. We performed pretreatment experiments in which intraperitoneal (i.p.) CDDO-Im or vehicle was administered daily from days -1 to +2 in relation to i.t. zymosan, and lungs were harvested on day +3. The mean percentage of lung parenchyma involved by inflammatory cell infiltration was significantly greater in the zymosan plus vehicle group compared to the zymosan plus CDDO-Im group (34±5% vs. 13±7%, respectively; p = 0.01), based on blinded review (Figure 7A and B). Consistent with these findings, BALF neutrophils were significantly reduced following CDDO-Im treatment (Figure 7C). Cytokines shown to be increased in zymosan-treated p47*^phox−/−^* mice compared to WT mice, including TNF-α, IL-17, and G-CSF, were reduced in BALF by CDDO-Im treatment (Figure 7D). In addition, BALF levels of IL-23 (an inducer of Th17 cell expansion) and LIX (an IL-17-stimulated chemokine [36]) were reduced by CDDO-Im treatment. We also performed experiments to measure the impact of CDDO-Im treatment on zymosan-induced NF-κB activation in p47*^phox−/−^*/HLL mice. By bioluminescence imaging, no differences were identified in NF-κB dependent luciferase activity in the lungs of p47*^phox−/−^*/HLL mice up to 3 days after zymosan plus CDDO-Im compared to the zymosan plus vehicle group (Figure 7E). In separate experiments, we found that CDDO-Im did not affect zymosan-induced NF-κB activation in BMDM from p47*^phox−/−^*/HLL mice (data not shown).

As an indicator of Nrf2 activity *in vivo*, we measured protein expression of NQO1 in lung homogenates. Consistent with *in vitro* findings, no differences in NQO1 expression were found between p47*^phox−/−^* mice with or without zymosan treatment; however, CDDO-Im administration significantly increased NQO1 protein levels in lungs of zymosan-treated p47*^phox−/−^* mice (Figure 7F and G). Similar results were obtained using quantitative RT-PCR to measure NQO1 mRNA expression (data not shown). In addition, Nrf2 activity was increased by CDDO-Im (Figure 7H), which, together with augmented NQO1 expression, shows the effectiveness of this treatment for *in vivo* Nrf2 activation.

**Figure 8:**
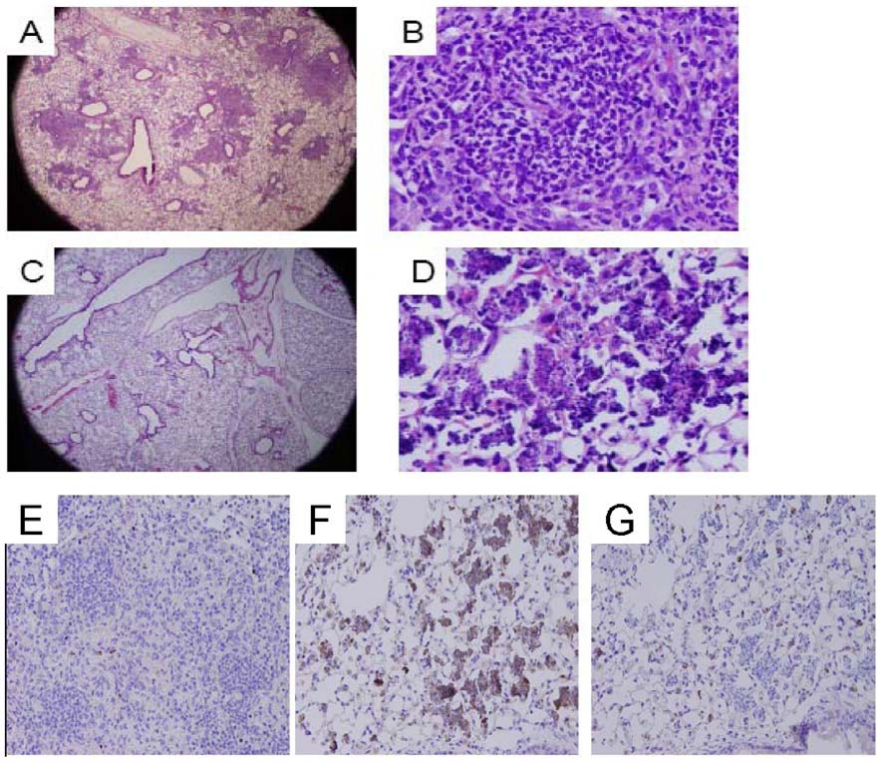
CDDO-Im reduces zymosan-induced lung inflammation in p47*^phox−/−^* mice in therapeutic studies by induction of apoptosis. CDDO-Im (i.p. 0.2 mg/mouse) or vehicle was administered daily from days 2 to 5 and lungs were harvested on day 6 in relation to i.t. zymosan administration. A) Lung section of a p47*^phox−/−^* mouse administered zymosan and vehicle shows well-defined granulomatous lesions occupying approximately 40% of the lung (H&E, 20x). B) Higher magnification (400x) shows dense cellular granulomata composed of neutrophils and lymphohistiocytic infiltrates in mouse treated with zymosan and vehicle. C) In contrast, scant areas of inflammation were present in the lungs of p47*^phox−/−^* mice administered zymosan and CDDO-Im (H&E, 20x). D) At higher magnification (400x), small foci of degraded inflammatory cells were observed in CDDO-Im treated p47*^phox−/−^* mice. E–G) Cleaved caspase-3 immunostaining was augmented in lungs of p47*^phox−/−^* mice administered zymosan and CDDO-Im compared to zymosan and vehicle. E) Lung section of zymosan and vehicle-treated mouse shows dense inflammatory lesions with occasional apoptosis (H&E, 200x). F) Lung section of zymosan and CDDO-Im-treated mouse shows sparse areas of inflammation composed of apoptotic cells that are positive (brown staining) for cleaved caspase-3 (H&E, 200x). G) Same section as B with rabbit isotype shows background staining within alveolar epithelial cells, but not in areas of inflammation (H&E, 200x). Addition of blocking peptide eliminated anti-cleaved caspase-3 staining with the exception of background activity, confirming specificity of staining (data not shown). Sections are representative of 4 p47*^phox−/−^* mice per treatment group.

In additional studies, we used a ‘therapeutic’ model to evaluate the effects of CDDO-Im on established lung inflammation in p47*^phox−/−^* mice. In these studies, i.p. CDDO-Im or vehicle was administered daily from days +2 to +5 in relation to i.t. zymosan, and lungs were harvested on day +6. Pyogranulomatous lesions involving approximately 40% of the lung, characterized by a central collection of neutrophils surrounded by lymphohistiocytic inflammation, occurred in mice that received zymosan and vehicle (Figure 8A–D). In contrast, mice administered zymosan and CDDO-Im had inflammatory infiltrates limited to <10% of the lung. The remaining areas of infiltrate in zymosan-treated mice in the CDDO-Im group contained pyknotic cells and inflammatory debris, with few viable cells. These apoptotic inflammatory cells were positive for cleaved (activated) caspase-3 immunostaining (Figure 8E–G), suggesting that CDDO-Im treatment induces inflammatory cell apoptosis when given after the onset of inflammation in p47*^phox−/−^* mice. Taken together, these results show p47*^phox−/−^* mice can be rescued from hyper-inflammatory responses by pharmacological NADPH oxidase-independent interventions targeting the Nrf2 pathway.

### Nrf2-Deficient Mice Have an Inflammatory Response Intermediate between WT and p47*^phox−/−^* Mice

**Figure 9:**
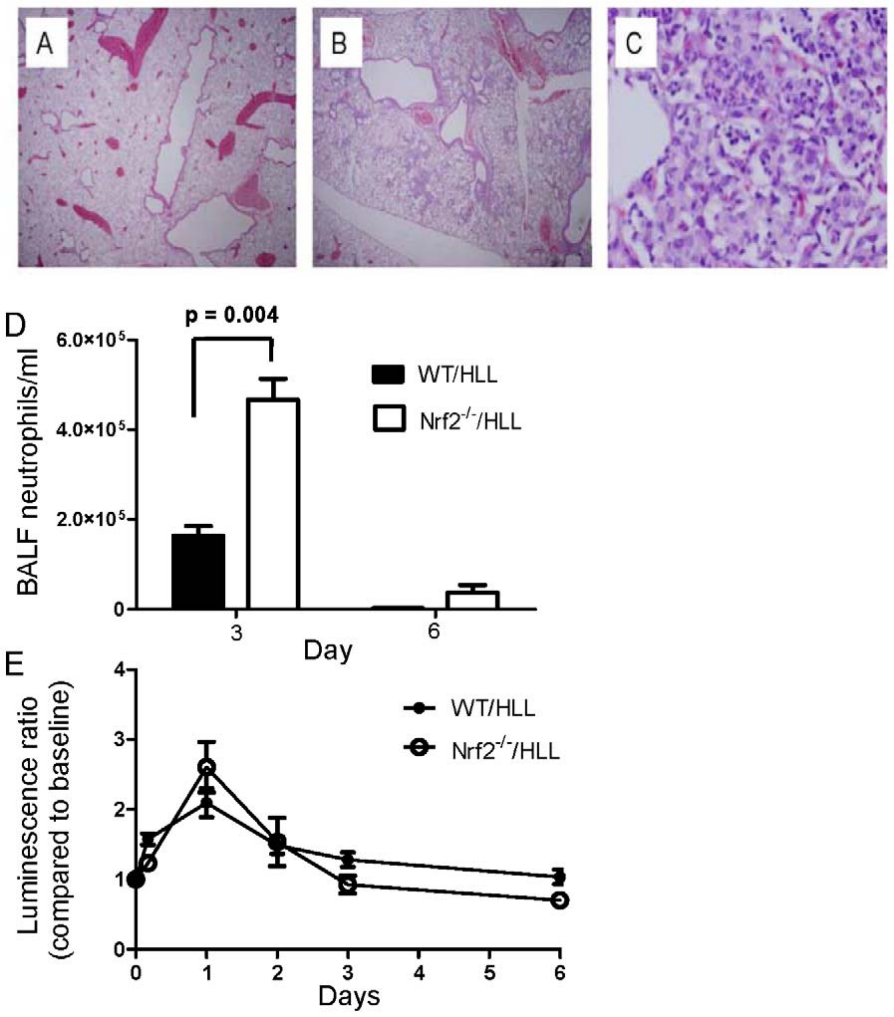
Nrf2^−/−^ mice develop increased i.t. zymosan-induced inflammation compared to wild type (WT) mice. A) Representative lung of WT mouse at day 3 after zymosan showing no inflammation (H&E, 20x). B) Nrf2^−/−^ mouse lung showing approximately 15% of lung involved with inflammatory infiltrates at day 3 after zymosan (H&E, 20x). C) Higher power magnification (200x) showing a predominantly histiocytic alveolar infiltrate with scattered foci of neutrophils and apoptotic cells in the lung of an Nrf2^−/−^ mouse. The above is representative of 3 mice per genotype. D) BALF neutrophilic leukocytosis was significantly greater in Nrf2^−/−^ versus WT mice at 3 days after zymosan. By day 6, the BALF neutrophil count was similar in both genotypes. There was no significant difference in the BALF macrophage concentration. E) Zymosan-induced lung NF-κB activation was similar in WT/HLL (n = 6) and Nrf2^−/−^/HLL (n = 7) mice (Two-way ANOVA, p = NS).

As Nrf2 activity is critical for the suppression of hyper-inflammatory responses and our studies indicated that Nrf2 is activated by NADPH oxidase *in vivo*, we asked whether Nrf2^−/−^ mice would have a hyper-inflammatory phenotype similar to p47*^phox−/−^* mice. Nrf2^−/−^ mice were crossed with NF-κB reporter (HLL) mice and compared to WT/HLL reporter mice after administration of zymosan. In Nrf2^−/−^/HLL mice, histological evidence of lung inflammation was more prominent than in WT/HLL mice at day 3 after zymosan (Figure 9A–C). In Nrf2^−/−^/HLL mice, the neutrophilic influx in BALF was significantly greater than in WT/HLL mice at day 3 after zymosan, but abated by day 6 (Figure 9D). Lung NF-κB activation was similar between Nrf2^−/−^/HLL and WT/HLL mice (Figure 9E). These results suggest that Nrf2 is primarily required for early control of zymosan-induced inflammation and does not appear to affect NF-κB activation. Taken together, these results point to activation of Nrf2 as being one, but not the only, pathway by which NADPH oxidase regulates inflammation.

Since CDDO-Im can affect both Nrf2-dependent and -independent pathways [37], we asked whether CDDO-Im would dampen inflammation in Nrf2−/− mice. Histological lung inflammation was similar at day 3 after i.t. zymosan administration in Nrf2−/− mice that received i.p. CDDO-Im (n = 7) versus vehicle (n = 8) from days −1 to +2 in relation to zymosan (data not shown), lending support to the notion that CDDO-Im attenuates inflammation in CGD mice primarily through Nrf2 activation.

### NADPH Oxidase Regulates Nrf2 and NF-κB Activation in Human Peripheral Blood Mononuclear Cells

**Figure 10:**
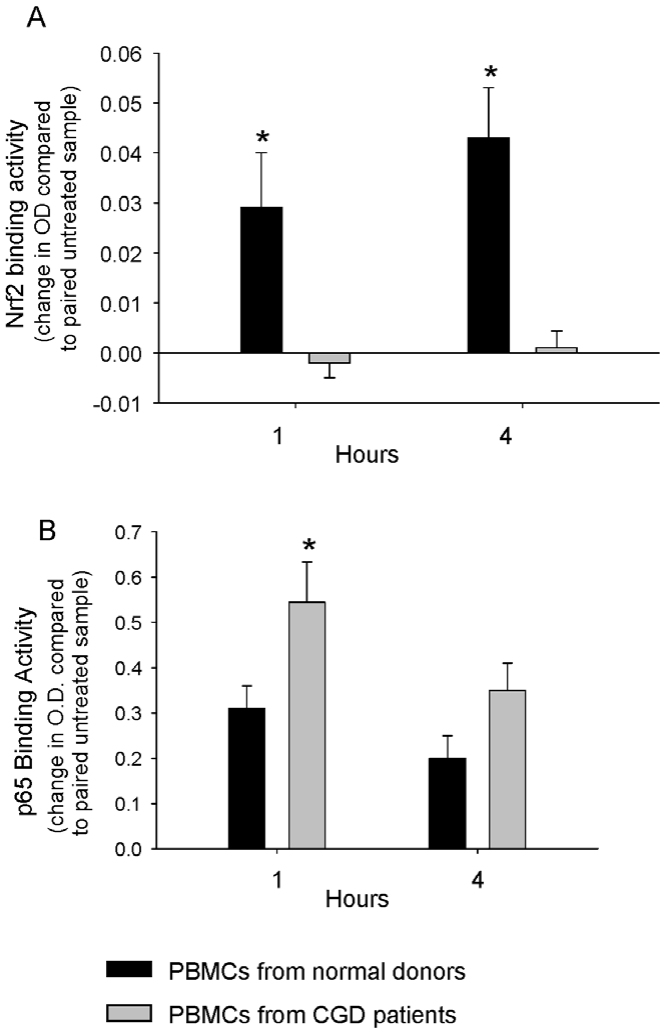
NADPH oxidase augments Nrf2 activation and restrains NF-κB activation in human PBMCs. PBMCs from normal donors (n = 8) and X-linked CGD patients (n = 5) were stimulated with zymosan (20 µg/ml). A) Nrf2 activation. B) NF-κB activation. *, p<0.05 comparing normal donor and CGD PBMCs.

We next asked whether NADPH oxidase influences Nrf2 and NF-κB activation in human cells by studying purified peripheral blood mononuclear cells (PBMCs) from normal donors and X-linked CGD patients. As shown in Figure 10A, zymosan-induced Nrf2 activation was uniformly defective in CGD PBMCs. In contrast, NF-κB activation from the same nuclear extracts was augmented in zymosan-stimulated CGD PBMCs compared to normal donor PBMCs (Figure 10B). Thus, our studies using human PBMCs were consistent with mouse data, and further support a key role for NADPH oxidase in regulating signaling through Nrf2 and NF-κB pathways.

The principal function of NADPH oxidase is to generate ROIs and activate neutrophil granular proteases that kill invading pathogens. The second and less recognized function of NADPH oxidase is to counterbalance these early pro-inflammatory events to limit tissue injury. Indeed, our studies suggest that inflammation may not be passively self-limited and that activation of anti-inflammatory pathways is required to protect the host from excessive inflammation. Consistent with studies in mice, NADPH oxidase activates Nrf2 while restraining NF-κB activation in human PBMCs. We propose a model in which NADPH oxidase-derived ROIs play a central role in limiting microbial ligand-induced inflammation by interacting in parallel with redox-sensitive targets that regulate NF-κB and Nrf2 activation.

Our results in Nrf2^−/−^ mice are consistent with other studies demonstrating a hyper-inflammatory phenotype [29], including increased inflammatory responses and mortality after systemic LPS challenge [38], [39]. Activation of Nrf2 likely mitigates ROI-induced inflammation and cellular injury [40], [41], [42], [43], [44], [45]. While our data point to Nrf2 as an important anti-inflammatory regulator activated by NADPH oxidase, the intensity and duration of inflammation were greater in CGD compared to Nrf2^−/−^ mice, arguing that dysregulation of Nrf2-independent pathways also contribute to excessive inflammation in CGD. That CDDO-Im activates Nrf2 independently of NADPH oxidase and limits inflammation suggests that Nrf2 activation may be a promising therapeutic strategy in controlling inflammatory complications in CGD. CDDO-Im may also have Nrf2-independent anti-inflammatory effects [34], [37], [46], [47], [48]. Use of double knockout CGD x Nrf2−/− mice will clarify whether the anti-inflammatory effect of CDDO-Im is Nrf2-specific.

In addition to defective Nrf2 activation, failure to down-regulate NF-κB activation likely contributes to the exaggerated inflammatory response in CGD. Zymosan and LPS treatment result in a marked increase and prolongation of NF-κB activation in the lungs of p47*^phox−/−^* mice and in isolated macrophages. CDDO-Im treatment increased lung Nrf2, but not NF-κB, activation, and NF-κB activation was not altered in zymosan-treated Nrf2−/− mice. Together, these findings suggest that NADPH oxidase regulates Nrf2 and NF-κB signaling in parallel to restrain inflammatory signaling induced by PRR activation. Other studies show the potential for cross-regulation of NF-κB and Nrf2 pathways [49], [50]. Indeed, it was recently shown that Keap1 binds to, and is required for, Cullin 3-based ubiquitination of IKKbeta, leading to downregulation of NF-κB [51].

Our findings are consistent with prior studies of human CGD PMBCs in which expression of NF-κB-dependent pro-inflammatory cytokines was increased following treatment with LPS [10], [11]. In addition, CGD mice have greater cigarette smoke-induced lung injury and NF-κB activation compared to WT mice [18]. Using acatalasemic mice, Zmijewski et al. [52] showed that H_2_O_2_ reduced NF-κB activation in neutrophils and diminished LPS-induced lung injury in mice. In contrast, we previously reported that early activation of NF-κB was reduced in the lungs of CGD mice treated with i.p. LPS [53] compared to wildtype mice, although inflammation was similar. The difference in results between our previous study [53] and the current work may be related to the route of LPS administration (i.p. versus i.t. in the current study), time of measurement of lung NF-κB activation in relation to LPS administration (90 minutes versus later time points in the current study), and the assay used to measure *in vivo* NF-κB activation (gel shift assay, which measures the presence of NF-κB dimers in the nucleus, versus use of HLL reporter mice to measure NF-κB-driven transcriptional activity in the current study). Other previous studies found that hepatic NF-κB activation and injury were reduced in CGD compared to WT mice after exposure to toxins [54], [55]. Together, these results underscore the complexity of the interaction of ROIs and NF-κB activation that is likely influenced by the specific stimulus, site of inflammation, kinetics of ROI generation, and numerous redox-sensitive targets that can activate [56] or inactivate [57] NF-κB.

In summary, our studies elucidate an under-appreciated role for ROIs in termination of innate immune responses through regulation of crucial intracellular signaling pathways. We and others have previously shown specific roles for NADPH oxidase in regulating neutrophil-endothelial cell interactions [58] and dendritic cell and T-cell phenotypes [16], [59], [60], [61], [62]. Romani et al. [16] demonstrated a central role of NADPH oxidase in determining the balance between Th17 and regulatory T-cell development through activation of tryptophan catabolism. These studies together with our current results show that NADPH oxidase calibrates immune homeostasis at multiple levels. Therefore, it is not surprising that attempts at antioxidant therapy to reduce tissue inflammation and injury have been unsuccessful. Manipulation of critical oxidant-regulated signaling pathways, like Nrf2, may be a more promising approach for prevention and treatment of inflammatory lung injury [63] and other disorders of inflammation. In addition, treatments that enhance Nrf2 activity could be beneficial for treating inflammatory complications of CGD.

## Materials and Methods

### Mice

Mice with a targeted disruption of the p47*^phox^* gene have a defective NADPH oxidase, rendering phagocytes incapable of generating measurable superoxide [20]. p47*^phox−/−^* mice were derived from C57BL/6 and 129 intercrosses, and were backcrossed 14 generations in the C57BL/6 background. Age and sex-matched C57BL/6 WT mice were used as controls. Nrf2^−/−^ mice were generated from C57BL/6 and 129 intercrosses as previously described [64], and were backcrossed 9 generations in the C57BL/6 lineage. Inbred C57BL/6 mice were used as WT controls. X-linked (gp91*^phox^*-deficient) mice were generated by Pollack et al. [21] We crossbred p47*^phox−/−^* mice with NF-κB HIV-LTR/luciferase (HLL) reporter mice (p47*^phox−/−^* /HLL) as previously described [23]. Nrf2^−/−^/HLL mice were derived in a similar fashion. This system enables visualization of NF-κB activation longitudinally at the level of the whole mouse and isolated organs and cells. WT and p47*^phox−/−^* bone marrow-derived macrophages (BMDMs) from these mice were generated as previously described [23]. Mice were maintained under specific pathogen free conditions and all procedures performed on animals were approved by the Institutional Animal Care and Use Committee at Roswell Park Cancer Institute and Vanderbilt University, and complied with all state, federal, and NIH regulations.

### Intratracheal Zymosan Administration

Zymosan (Sigma, St. Louis, MO) was diluted to a concentration of 2.5 mg/ml in saline, sonicated until the particles were suspended homogenously and frozen at −20°C. Prior to use, the zymosan stock was diluted to 0.8 mg/mL and autoclaved to ensure sterility. Mice were anesthetized with i.p. injections of Avertin (380 mg/kg). Mice were restrained, hair plucked from the throat and the area cleansed with betadine and alcohol. A medial cut was made in the skin above the trachea followed by a medial cut in the tracheal sheath. A BD Insyte (BD, Franklin Lakes, NJ) cannula was inserted into the trachea just above the bifurcation and 25 ul of the zymosan suspension followed by 25 µl of air were injected. The incision was closed with a suture. Mice were given 1 ml of sterile PBS IP for re-hydration, placed on a heating pad and monitored for recovery.

### Bioluminescence Imaging

Mice were anesthetized, received 1 mg of D-luciferin retro-orbitally, and were imaged as described previously [23], in an IVIS cooled charged coupled device (Xenogen Corporation, Alameda, CA). Data were collected and analyzed using Living Image v.2.50 (Xenogen Corporation, Alameda, CA) and IgorPro (Wavemetrics, lake Oswego, OR) software.

### CDDO-Im (1-[2-cyano-3-,12-dioxooleana-1,9(11)-dien-28-oyl]imidazole)

CDDO-Im is a semi-synthetic triterpenoid that potently induces Nrf2 activity[34]. For each experiment, CDDO-Im was dissolved in a 10% DMSO 10% cremaphor-EL PBS solution as previously described [39]. Intraperitoneal CDDO-Im (0.2 mg/mouse per day) or vehicle was administered at different times in relation to zymosan challenge.

### Cytokine and Chemokine Measurement

Mice were killed by CO_2_ asphyxiation, and bronchoalveolar lavage (BAL) was performed using a total of 2 ml of cold saline per animal. BALF IL-23 and LIX (a neutrophil chemokine) were assessed by ELISA per the manufacturer's instructions (eBioscience, San Diego, CA and R&D Systems, Minneapolis, MN, respectively). All other cytokines and chemokines were measured at the Roswell Park Cancer Institute core facility flow cytometry laboratory using the multiplexed flow cytometry assay in which several cytokines can be quantitated simultaneously on the same sample. The Luminex 100 platform is used to acquire data from soluble bead arrays in which each bead set has a separate capture reagent attached to the surface that is directed against a single cytokine or chemokine.

### Lung Histopathology

After sacrifice and bronchoalveolar lavage, mouse lungs were infused with 10% neutral buffered formalin via the trachea. Parrafin-embedded sections were prepared and stained with hematoxylin and eosin (H&E). The percentage of lung involved by granulomatous or consolidative inflammation was scored in each mouse as follows: 0%, 5%, 10%, and then by 10% increments (e.g., 20%, 30%, 40%, etc.). The predominant inflammatory cell type was scored as neutropilic or lymphohistiocytic. Histopathology was assessed by one of us (BHS) in a blinded fashion.

To evaluate for apoptosis, immunohistochemistry to detect cleaved caspase-3 on paraffin-embedded sections was performed. Activation of caspase-3 requires proteolytic cleavage of its inactive zymogen. Cleaved caspase-3 (Asp175) polyclonal antibody (Cell Signaling, Danvers, MA) detects the large fragment of activated caspase-3 resulting from cleavage adjacent to Asp175. The cleaved caspase-3 antibody (0.33 µg/ml) was applied to slides for 1 hour. Rabbit IgG isotype and blocking peptide were used as specificity controls. A biotinylated secondary anti-rabbit antibody followed by the Elite ABC reagent (Avidin: Biotinylated enzyme Complex) and diaminobenzidine-based peroxidase substrate were added per the manufacturer's instruction (Vector Labs, Burlingame, CA), and counterstained with hematoxylin. The color reaction product results in brown staining.

### Bone Marrow Transplantation

Donor mice were sacrificed using cervical dislocation to ensure the collection of unadulterated material. Next, femurs and tibias were removed, the tips excised, and the bone marrow flushed out using cold 0.1% BSA solution in PBS. Bone marrow cells were then washed and resuspended in cold PBS. Recipient mice received myeloablative conditioning with 12Gy of ^137^Cs γ total body irradiation (0.74Gy/min) split 6Gy a day for two days. Eight hours after the final radiation dose, mice were injected in the tail vein with the donor bone marrow cells (1×10^6^ cells per recipient mouse). Recipient mice were administered i.t. zymosan at 31 days after transplant. Seven days later, peripheral blood and lungs were harvested. NADPH oxidase activity in peripheral neutrophils of transplanted mice was evaluated using the fluorescent probe, dihydrorhodamine 123 (DHR), as previously described [65].

### Nuclear Protein Extraction

At the indicated time points, samples were placed on ice, washed with PBS, and cytoplasmic and nuclear extracts were obtained using the NE-PER kit (Pierce, Rockford, IL) in the presence of Complete mini, EDTA-free protease inhibitor cocktail (Roche Diagnostics, Switzerland) at 4°C. Then samples stored at −80°C.

### Analysis of Nrf2 Activation

Western blot analysis of nuclear protein fractions was performed by Odyssey system (LI-COR Bioscience, Nebraska USA), using antibodies specific for Nrf2, TBP, beta-actin (Santa Cruz Technology), NQO1 (Cell Signaling Technology). In whole lung nuclear protein extracts, Nrf2 was measured by the TransAM Nrf2 ELISA (Active Motif, Carlsbad, CA) using the manufacturers instructions.

### PBMCs from CGD Patients and Normal Donors

Whole blood from X-linked CGD patients and normal donors were collected at the NIH Clinical Center in heparinized tubes and processed immediately. Serum was obtained from a distinct healthy donor in serum separator tubes, aliquoted, and frozen at −20°C until ready for use. Cell culture media was prepared from RPMI 1640 supplemented with 2 mM l-glutamine, 100 U of penicillin/mL, 100 µg streptomycin/mL, 10 mM HEPES buffer, and 10% donor serum, and filter sterilized. Zymosan (Sigma) was re-suspended in sterile filtered sodium chloride 0.85% (Quality Biologicals), autoclaved, and kept at 4°C until ready for use. Whole blood from each donor was diluted in a 1∶1 ratio with HBSS without divalent cations. PBMCs were collected following standard separation on Lymphocyte-Separation medium (Mediatech). The PBMC pellet underwent hypotonic lysis with ACK Lysing buffer (Quality Biologicals) to remove erythrocytes, then washed twice with HBSS and re-suspended in cell culture media. PBMCs were enumerated by hemacytometer and confirmed >95% viable by trypan blue exclusion. PBMCs from each subject were inoculated in duplicate into 6 well plates at 3×10^6^ cells/mL, allowed to adhere for 2 hours at 37°C in 5% CO_2_, then stimulated with zymosan (20 µg/ml), and incubated at 37°C in 5% CO_2_. After 1 and 4 hours of stimulation, cell culture medium was removed, each well was washed twice in phosphate-buffered saline, and the plate was frozen at −80°C until ready for nuclear protein extraction. Nuclear extracts were isolated, and Nrf2 activity was measured by the TransAM Nrf2 ELISA, using the same methods as mouse samples. NF-κB activation in nuclear extracts was measured by relA (p65) DNA binding using the TransAM NF-κB ELISA kit. All subjects gave written informed consent for research testing under approved protocols of the NIH.

### Statistics

Prism software was used for statistical analysis and to display graphical data. Inter-group comparisons were made using the non-parametric Mann-Whitney method. A p-value of <0.05 was considered statistically significant. For time-course experiments, two-way ANOVA with Bonferroni post-testing at individual time points was used.
